# Tail—aware heterogeneous graph neural networks for multi—class drug—drug interaction prediction

**DOI:** 10.1093/bioinformatics/btag413

**Published:** 2026-08-21

**Authors:** Varshini Venkatesh, Varsha G, Dhannya S M

**Affiliations:** Department of Computer Science and Engineering, Sri Sivasubramaniya Nadar College of Engineering, OMR, Chennai, Tamil Nadu 603110, India; Department of Computer Science and Engineering, Sri Sivasubramaniya Nadar College of Engineering, OMR, Chennai, Tamil Nadu 603110, India; Department of Computer Science and Engineering, Sri Sivasubramaniya Nadar College of Engineering, OMR, Chennai, Tamil Nadu 603110, India

## Abstract

**Motivation:**

Polypharmacy is increasingly common in clinical practice, and the sheer number of possible drug combinations makes manual interaction screening impractical. Early computational approaches relied on chemical similarity metrics and rule-based systems, while subsequent machine learning and deep learning methods improved predictive power but continued to treat drugs as isolated entities, missing the broader biological context that governs interaction behaviour. Graph Neural Network (GNN) based methods address this by modeling drugs alongside proteins, diseases, and side effects in a shared relational graph, but tend to fall short on sparsely represented long-tail interaction classes due to the severe class imbalance that characterizes real-world biomedical interaction data.

**Results:**

We construct a large-scale heterogeneous biomedical knowledge graph—26 408 nodes across five entity types and 1 679 387 edges across six relation types—and benchmark MLP, GCN, HGT, and RGCN for 105- class DDI prediction. RGCN achieves the strongest overall performance (Macro F1: 0.694, Recall: 0.720), with relation-specific weight matrices proving the critical factor in heterogeneous DDI modelling. Among class imbalance strategies tested, Tail-Aware Focal Loss outperforms standard cross-entropy by 3.1% on Macro F1 and 7.4% on Recall, striking a better balance between class performance than either weighted cross-entropy or weighted random sampling.

## 1 Introduction

Drug-drug interactions (DDIs) remain one of the most persistent sources of preventable harm in clinical medicine. When two or more drugs are co-administered, their combined pharmacological effects can deviate substantially from what either agent would produce in isolation—sometimes beneficially, but more often in ways that are difficult to anticipate. The consequences span a wide spectrum, from mild tolerability issues to serious adverse events including cardiac arrhythmias, abnormal bleeding, and acute organ failure. This problem has grown considerably worse as prescribing complexity has increased. Polypharmacy—broadly defined as the concurrent use of five or more medications—is no longer an edge case. Among elderly patients managing chronic conditions, regimens involving ten or more drugs are routine, and the combinatorial interaction space this creates is beyond what any clinician can track reliably without computational assistance.

For much of the past few decades, the primary mechanisms for detecting DDIs have been clinical trials and post-market pharmacovigilance. Both are indispensable, but both are fundamentally reactive—they identify problems after drugs have already reached patients. Clinical trials are not designed to probe interaction effects systematically, and post-market surveillance can take years to surface a signal. There is a genuine unmet need for methods that flag potential interactions prospectively, before prescriptions are written.

Early computational approaches framed DDI prediction as pairwise classification using chemical similarity metrics (Vilar *et al.* 2014). These methods formalised existing clinical knowledge rather than generalising beyond it. Machine learning followed, applying learned models to molecular feature vectors (Thomas* et al.* 2017), and deep learning extended this through architectures such as DeepDTA (Öztürk *et al.* 2018) and TransformerCPI (Chen *et al.* 2020), achieving substantial gains in predictive power. Even so, drugs continued to be evaluated in isolation—without modelling the relational biological web of shared enzymatic targets, overlapping pathways, and competing disease mechanisms that determines how interactions actually manifest in vivo.

Graph Neural Networks (GNNs) addressed this directly. By encoding drugs, proteins, diseases, and side effects as nodes in a shared relational graph, GNN—based models learn representations that reflect a drug’s full biological context. Decagon (Zitnik *et al.* 2018), SumGNN (Yu *et al.* 2020), and KGNN (Lin *et al.* 2020) demonstrated convincingly that this approach substantially outperforms earlier methods on standard DDI benchmarks. That said, a critical gap remains. Real-world DDI databases exhibit a pronounced long-tail structure: a small number of heavily studied drug pairs account for the vast majority of annotated interactions, while newer and less commonly prescribed agents appear sparsely (Liu *et al.* 2021). Standard cross-entropy optimisation is dominated by majority classes, starving minority classes of meaningful gradient updates (Cao *et al.* 2019)—a failure mode well understood in the imbalanced learning literature (Lin *et al.* 2017) but underaddressed in DDI modelling. Models that perform reliably on frequent interaction types while failing on the tail are of limited clinical value, since it is precisely the rare interactions that carry the greatest patient safety risk.

This study presents two contributions. First, we construct a large-scale heterogeneous biomedical knowledge graph from nine curated databases, yielding 26 408 nodes and 1 679 387 edges across six relation types, and conduct a rigorous comparative evaluation of six GNN architectures for 105-class DDI prediction, isolating the contribution of relational structure and heterogeneous message passing to model performance. Second, we systematically evaluate three strategies for handling the severe class imbalance inherent in biomedical interaction data, introducing a Tail-Aware Focal Loss formulation that combines focal modulation with effective sample size weighting, and demonstrate that it achieves the best balance between class performance of all strategies tested.

## 2 Methodology

### 2.1 Graph construction

#### 2.1.1 Data sources and integration

The heterogeneous graph integrates DrugBank (Wishart *et al.* 2018) for canonical drug entities and documented drug-drug and drug-protein links. PubChem (Kim *et al.* 2025) supplies standardized structures for drug featurization. Drug-protein interactions are retrieved from ChEMBL (Zdrazil *et al.* 2024) and BioSNAP (Zitnik *et al.* 2018). UniProtKB (The UniProt Consortium 2023) is used to normalize protein entities to UniProt IDs and retrieve corresponding sequences. STRING (Szklarczyk *et al.* 2023) contributes PPIs with confidence score above 950 to capture pathway-level context. To enrich the graph with clinical and pharmacological context beyond core molecular interactions, DRKG (DRKG Team 2020) was used as the structural backbone for auxiliary connections, supplying the fundamental drug-disease and semantic associations. This baseline was systematically extended by integrating data from SIDER (Kuhn *et al.* 2016) and the WHO ATC system (World Health Organization 2025).

**Figure 1:**
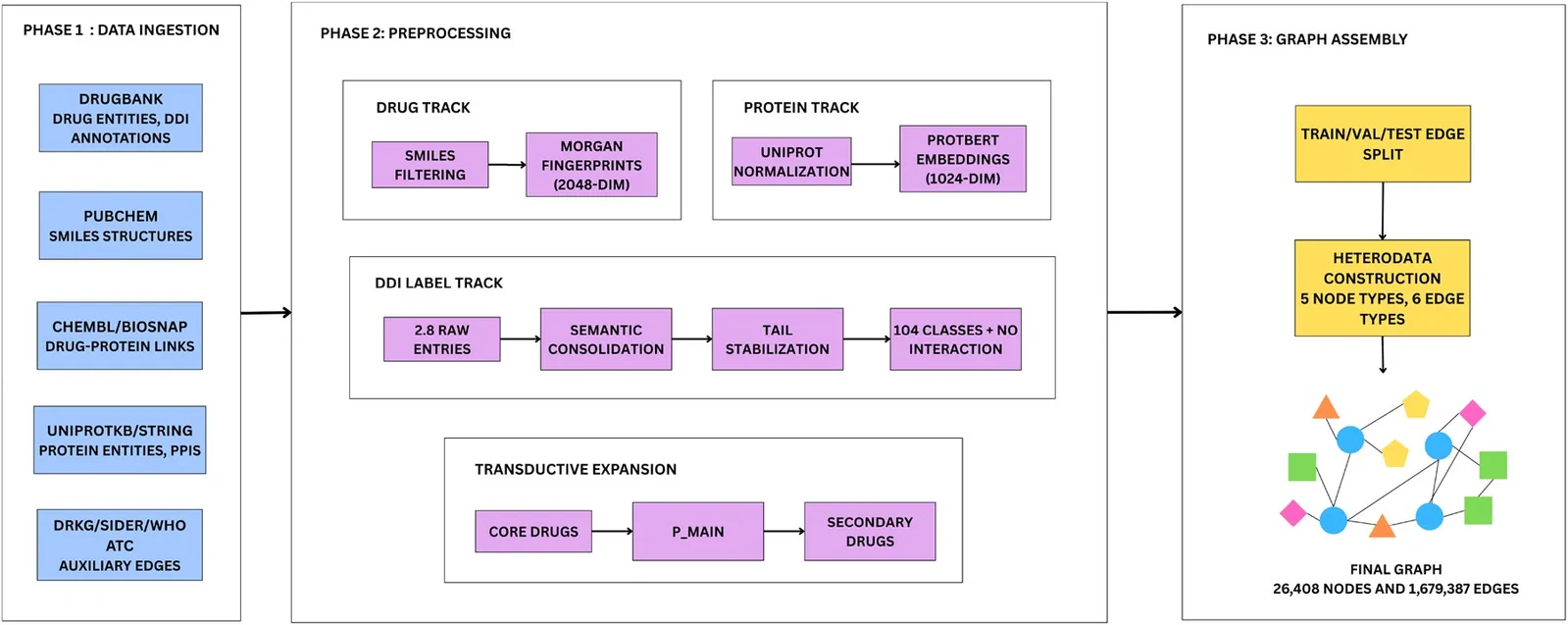
Overview of the heterogeneous graph construction pipeline. Raw data from nine curated biomedical databases are ingested, preprocessed through drug featurization, protein encoding, and DDI label normalization, and assembled into a unified HeteroData graph comprising 26 408 nodes and 1 679 387 edges across five node types and six relation types.

A unified drug set was synthesized by merging DrugBank and ChEMBL entries using CompoundDB4J (Murali *et al.* 2020). A filtering process retained only drugs with valid SMILES (Weininger 1988) representations. To capture indirect biological pathways without computational bloat, a transductive expansion strategy was employed: (1) core drugs with known interactions were identified; (2) proteins directly targeted by these drugs ($P_{\mathrm{main}}$) were added; (3) the graph was then expanded to include secondary drugs targeting $P_{\mathrm{main}}$ and additional proteins linked to $P_{\mathrm{main}}$ via high—confidence PPIs. Although STRING recommends 700 as a conservative cutoff for downstream computational analyses, higher thresholds reduce noisy or weakly supported associations at the cost of producing smaller networks biased toward well-studied proteins (Szklarczyk *et al.* 2023). This trade-off is acceptable here because the transductive expansion strategy already anchors the network to drugs with known interactions and their primary targets. Lower-confidence PPIs would mainly introduce peripheral proteins that increase message-passing overhead without contributing meaningful interaction-relevant signal. Auxiliary nodes—ATC codes, diseases, and side effects were added to provide additional clinical and pharmacological context beyond core molecular interactions. Only curated relationships with database support were included. Drug-ATC edges represent therapeutic classifications, drug-disease edges represent known treatment associations, and drug-side effect edges represent documented adverse effects. To keep the graph relevant and connected, auxiliary nodes were included only if they were linked to drugs present in the final drug set. Isolated nodes with no valid drug connections were removed, preventing disconnected components that contribute no useful message-passing signal. No missing relationships were inferred; every auxiliary edge in the final graph corresponds to a documented biomedical association. The full data integration and preprocessing pipeline is illustrated in Fig. 1. The DDI annotations required substantial preprocessing before they could be used as supervised labels. DrugBank interaction descriptions are written as unstructured free text with inconsistent phrasing and synonym variation, making direct use as class labels infeasible. To address this, 2.8 million raw entries were parsed into discrete *(mechanism, action)* tuples and normalized through a three-stage pipeline. For example, the raw XML description:

Diazepam may increase the sedative and respiratory depressant activities of Morphine

Was first standardized through synonym replacement and entity masking:

DRUG may increase the sedative and respiratory depressant activities of DRUG

This was then mapped into the *(mechanism, action)* tuple *(sedative and respiratory depressant activities, increase)*.

The first normalization stage performed atomic decomposition into: *increase sedation* and *increase respiratory depression*.

The second stage consolidated semantically equivalent terms into canonical labels using a manually curated synonym dictionary. For example, *drowsiness*, *somnolence*, and *sedation* were unified under the canonical label *increase sedation*.

The third stage addressed tail sparsity by grouping interaction classes with fewer than five instances into broader semantically coherent categories where appropriate.

These normalized labels were then used to construct DDI graph edges: *(Diazepam, Morphine, increase sedation)* and *(Diazepam, Morphine, increase respiratory depression)*.

Overall, this normalization pipeline reduced 2847 raw DrugBank interaction description variants into 104 canonical interaction classes plus one no-interaction class, yielding a clinically interpretable and statistically tractable 105-class prediction space.

This preprocessing distilled the linguistic noise into 104 distinct interaction classes plus one no interaction class yielding 105 classes, ensuring a statistically robust label space for supervised learning.

#### 2.1.2 Graph schema and feature generation

The heterogeneous graph is formally defined as G=(V,E). The node set *V* includes drugs, proteins, and auxiliary entities (diseases, side effects, ATC codes) whose features were initialized using domain-specific encoders to address data heterogeneity. Drug nodes were featurized as 2048-dimensional Morgan fingerprints (radius = 2) (Rogers and Hahn 2010), capturing local atomic neighbourhoods and functional groups. Protein sequences were encoded using the pre-trained ProtBERT model (Elnaggar *et al.* 2022); the mean-pooled representation of the final hidden layer was computed to generate 1024-dimensional embeddings representing each protein’s functional context. For auxiliary nodes, learnable embeddings were initialized and optimized jointly with GNN parameters via backpropagation.

The edge set *E* is defined by two distinct semantic roles. Auxiliary and biological associations—comprising Drug→Protein, Protein→Protein (PPI), Drug→Disease, Drug→SideEffect, and Drug→ATC interactions—are binary and unweighted, serving primarily to propagate message-passing signals across the graph. In contrast, Drug-Drug Interaction (DDI) edges are treated as labeled annotations for supervised learning; each edge $e_{ij}$ is assigned a class c∈{1,…,105}, representing a specific pharmacological interaction type.

**Figure 2:**
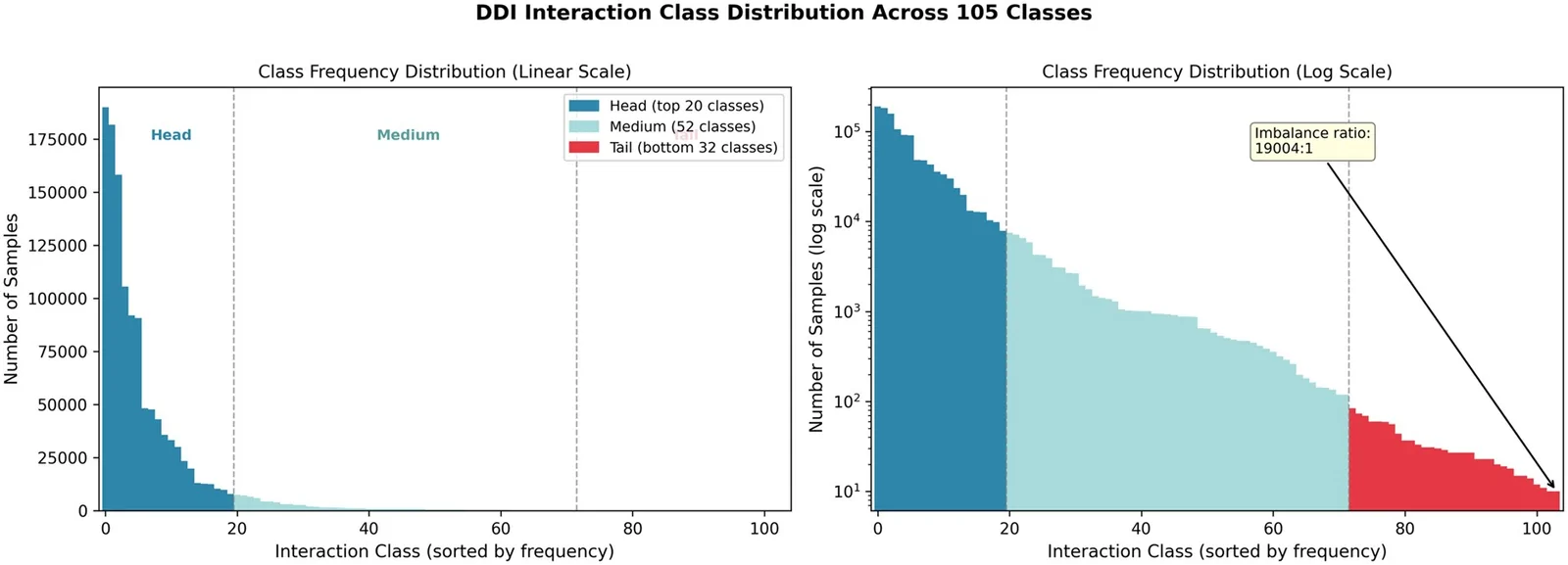
DDI interaction class distribution across 105 classes, sorted by frequency. *Left*: Linear scale reveals the sharp drop-off from head to tail classes. *Right*: Log scale exposes the full extent of the long-tail structure, with an imbalance ratio of 19 004:1 between the most and least frequent interaction types. Classes are grouped into head (top 20), medium (52), and tail (bottom 32) based on frequency thresholds.

The resulting DDI label distribution is highly skewed, as shown in Fig. 2. The top 20 head classes collectively account for the vast majority of annotated edges, while 32 tail classes are represented by very few samples each—with an overall imbalance ratio of 19 004:1 between the most and least frequent interaction types. This severe skew directly motivates the tail-aware loss functions evaluated in the Class Imbalance Handling section below.

The disparate biological layers are organized into a unified HeteroData structure (Fey and Lenssen 2019), which mirrors biological reality by permitting distinct computational rules for molecular binding versus clinical associations. To ensure rigorous evaluation, annotated DDI edges were partitioned into train, validation, and test splits; validation and test edges were strictly excluded from message-passing connectivity during training to prevent data leakage. Reverse edges were added to facilitate bidirectional message passing. Detailed node counts and edge type distributions are summarised in Table 1.

**Table 1:** Summary of the heterogeneous graph statistics.

Category	Entity/Relation	Count
*Node Types*		
Molecular	Drug	9495
	Protein	9816
Auxiliary	Side Effect	5698
	Disease	1181
	ATC Code	218
	**Total Nodes**	**26** **408**
*Edge Types*		
Molecular	Drug → Protein	2 29 542
	Protein → Protein	3 05 283
Auxiliary	Drug → Disease	5383
	Drug → Side Effect	1 38 521
	Drug → ATC	2892
Prediction	Drug → Drug (Train)	9 97 766
	**Total Edges**	**16** **79** **387**

DDI edges correspond to the training split only.

Edge counts shown for a single direction only; reverse edges are added during training for bidirectional message passing.

Bold lettering has been used for column titles and to represent grand total of different types of nodes and edges.

### 2.2 Graph neural network architectures

Six architectures were benchmarked under identical experimental conditions in order to systematically assess the contribution of relation-specific message passing and graph structure to DDI prediction. In order to guarantee that variations in performance are exclusively due to architectural decisions rather than experimental setup, all models use the same input features, training split, negative sampling technique, and edge MLP decoder.

#### 2.2.1 Multi layer perceptron

The MLP (Goodfellow *et al.* 2016) serves as the non-graph baseline. It takes the raw feature vectors of two drugs, concatenates them, and passes them through a series of fully connected layers to produce a class prediction. There is no message passing, no neighbourhood aggregation, and no graph structure involved whatsoever. Its purpose is to establish a lower bound—any improvement over MLP comes purely from the graph, not from the drug features themselves.

#### 2.2.2 Homogenous graph convolutional network

The homogeneous GCN (Kipf and Welling 2017) operates on a stripped down drug-only graph where the only edges present are known DDI links. It applies standard graph convolution, aggregating information from a drug’s direct DDI neighbours to update its representation. Because this version of the graph contains no proteins, diseases, or side effects, it isolates the contribution of graph structure alone—without any biological context from auxiliary nodes. This sits one step above MLP as a baseline.

#### 2.2.3 Heterogenous graph convolutional network

The heterogeneous GCN operates on the full graph—drugs, proteins, diseases, side effects, and ATC codes—but treats all edge types identically during message passing. There is no distinction between a drug-protein edge and a drug-disease edge; the same aggregation rule applies to everything. Comparing this against the homogeneous GCN directly shows how much auxiliary biological context helps, independent of any relational parameterization.

#### 2.2.4 Heterogeneous graph transformer

The Heterogeneous Graph Transformer (HGT) (Hu *et al.* 2020) extends standard graph attention to the heterogeneous setting by conditioning attention coefficients on both node and edge types. This allows the model to learn semantically distinct aggregation patterns for different relation types—for instance, treating drug-protein binding differently from drug-disease associations during message passing. HGT represents the attention-based approach to heterogeneous graph learning and provides a natural comparison point for evaluating whether explicit relational parameterization offers advantages over attention-based type differentiation.

#### 2.2.5 Relational graph convolutional network

**Figure 3:**
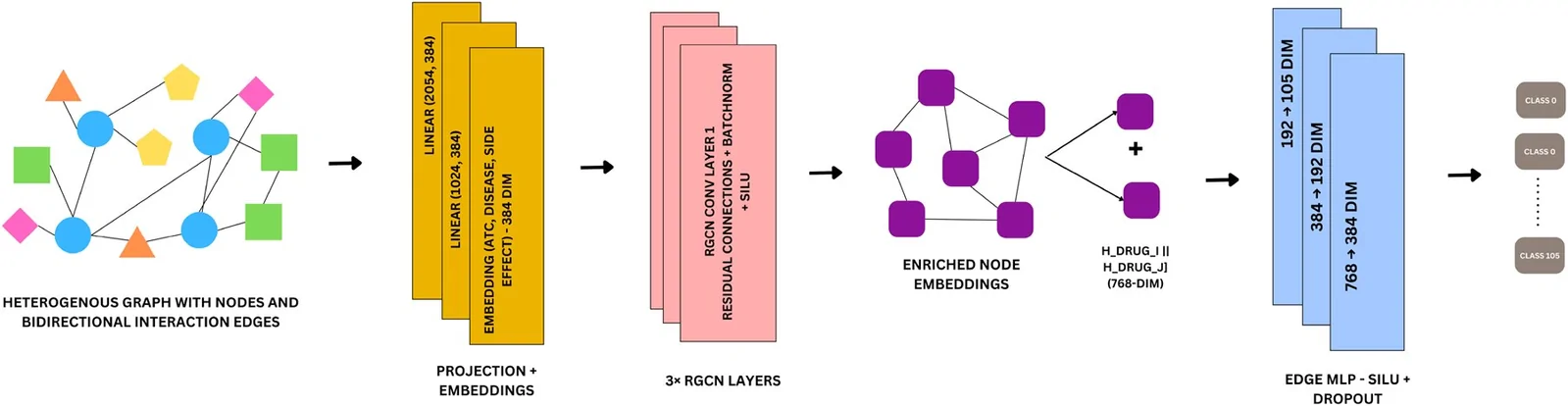
RGCN model architecture for DDI prediction. Node features from the heterogeneous graph are passed through three relational convolutional layers, each maintaining separate weight matrices per relation type to enable type—aware message aggregation. The resulting drug node embeddings are concatenated for each candidate drug pair and passed through an edge MLP decoder to produce a probability distribution over 105 interaction classes.

The Relational Graph Convolutional Network (RGCN) (Schlichtkrull *et al.* 2018) assigns a distinct weight matrix to each relation type in the graph, enabling type-aware message aggregation across all edges. Unlike HGT, which modulates attention scores based on type, RGCN applies entirely separate linear transformations per relation—a stronger form of relational inductive bias that may be better suited to settings where different edge types carry fundamentally different biological semantics. The RGCN architecture used in this work employs three relational convolutional layers followed by an edge MLP decoder for DDI classification, as illustrated in Fig. 3.

#### 2.2.6 Hybrid RGCN—HGT

The hybrid model is motivated by a straightforward observation—RGCN and HGT have complementary strengths. RGCN is good at capturing relational structure through type—specific transformations but collapses the heterogeneous graph into a flat node space to do so. HGT preserves the heterogeneous node—type structure throughout but relies on attention modulation rather than fully separate parameters. The hybrid runs both branches in parallel on the same projected input embeddings. The RGCN branch processes a flattened version of the graph with residual connections, while the HGT branch processes the full heterogeneous graph with type—aware attention. The outputs of both branches are concatenated per node and passed through a learned fusion layer that compresses them back into a single unified representation, which is then used for DDI classification.

### 2.3 Class imbalance handling

#### 2.3.1 Weighted cross—entropy loss

One of the most straightforward ways to address class imbalance is to make the model pay more attention to the classes it rarely sees. Weighted cross—entropy does exactly this—each class is assigned a weight inversely proportional to its frequency in the training set, so misclassifying a rare interaction type costs more than misclassifying a common one. Upweighting rare classes forces the model to attend to them during optimisation. It is a lightweight intervention—nothing about the training loop changes beyond passing a weight vector to the loss function (Cao *et al.* 2019)—which makes it a natural first step before reaching for more sophisticated approaches.

#### 2.3.2 Weighted random sampler

Instead of changing how the loss is computed, this strategy changes what the model sees during training. Drug pairs are sampled with a probability inversely proportional to their class frequency (Cui *et al.* 2019), which means rare interaction types appear in each batch far more often than they naturally would. The effect is a more balanced class distribution across training batches, without any actual data augmentation or duplication. The tradeoff, however, is that head classes get undersampled in the process—the model sees them less frequently, and while tail recall tends to improve, majority class performance can become noticeably unstable. This precision—recall tension shows up clearly in the results and makes weighted random sampling a less reliable choice when overall classification stability matters.

#### 2.3.3 Tail—aware focal loss

Focal loss was originally developed for dense object detection, where the sheer volume of easy background examples drowns out the gradient signal from the harder, more informative ones (Lin *et al.* 2017). The fix is to dynamically downweight well—classified examples during training using a modulating factor ${\left(1-p_{t}\right)}^{\gamma }$, which reduces the loss contribution of confident predictions and concentrates gradient signal on misclassified examples. The complete focal loss formulation is:


(1) $$L_{\text{focal}}=-\alpha_{t}{\left(1-p_{t}\right)}^{\gamma }\;\text{log}(p_{t})$$

Where $p_{t}$ is the model’s estimated probability for the true class and γ controls how aggressively easy examples are downweighted. In this work, γ is set to 2.0.

This is extended with effective sample size weighting, drawn from class—balanced loss theory (Cui *et al.* 2019). Rather than using raw class counts to compute weights, each class is weighted by its effective number of samples, defined as:


(2) $$E_{n}=\frac{1-\beta^{n}}{1-\beta }$$

Where *n* is the number of training examples for that class and β is a smoothing hyperparameter set to 0.99. This formulation diminishes the marginal benefit of adding more samples to already well-represented classes, producing a smoother and more stable weighting scheme than inverse frequency alone. The per-class weight is then computed as:


(3) $$\alpha_{c}=\frac{1-\beta }{1-\beta^{n_{c}}}$$

Which feeds directly into the $\alpha_{t}$ term in Equation 1. The combination of hard example mining through focal modulation and frequency correction through well suited to the 105—class DDI setting, where some tail classes have fewer than ten training examples and standard loss functions provide essentially no useful gradient signal for them. Tail-Aware Focal Loss also introduces negligible computational overhead—the modulating factor ${\left(1-p_{t}\right)}^{\gamma }$ and weights $\alpha_{c}$ are computed directly from existing logits with no additional forward passes, unlike weighted random sampling which incurs per-step batch resampling cost. This makes it the most practical strategy across both performance and efficiency.

## 3 Results

All models were trained and evaluated under identical conditions using an 80/10/10 train/validation/test split with a shared negative sampling strategy. Macro F1 is used as the primary comparison metric throughout, as it balances precision and recall equally across all 105 classes.

### 3.1 GNN architecture comparison

Table 2 summarises the performance of all six architectures evaluated on the heterogeneous graph for 105—class DDI prediction.

**Table 2:** Performance comparison of GNN architectures.

Graph	Model	Accuracy	Precision	Recall	F1
Homogeneous	GCN	0.8135	0.5985	0.6085	0.5915
Heterogeneous	MLP	0.7851	0.4813	0.4182	0.4216
	GCN	0.8965	0.6145	0.6459	0.6259
	HGT	0.9181	0.6397	0.6703	0.6185
	RGCN	**0.9521**	**0.6964**	**0.7196**	**0.6940**
	Hybrid RGCN—HGT	0.9481	0.6697	0.7103	0.6785

All metrics are macro—averaged across 105 classes.

Hybrid RGCN—HGT is included as an ablation.

Best results are highlighted in bold.

All architecture comparisons were conducted using standard cross-entropy loss. This was a deliberate choice—evaluating architectures under the baseline loss isolates the contribution of graph structure and relational parameterisation to performance, independent of any imbalance correction. Before interpreting these results, it is worth noting that accuracy alone is a misleading metric in this setting. With 105 classes and a severely skewed distribution, a model can achieve high accuracy simply by performing well on head classes while ignoring the tail entirely. Macro recall is arguably the most clinically meaningful metric—failing to detect a rare but dangerous interaction is a more serious failure than a false positive. Macro F1 balances precision and recall equally across all classes and is used as the primary comparison metric throughout.

The MLP baseline achieves a macro F1 of 0.4216, establishing an upper bound on what raw drug features alone can achieve. The graph structure is doing the heavy lifting—any improvement over MLP reflects information gained from neighbourhood aggregation rather than the features themselves.

Moving to the homogeneous GCN, which operates on a drug-only graph where edges represent known DDI links, lifts F1 to 0.5915. This is a meaningful step up, but the model has access to no biological context beyond the interaction graph itself—no proteins, no diseases, no side effects.

The jump to heterogeneous GCN is where things get interesting. F1 improves from 0.5915 to 0.6259 with the exact same architecture—the only change is the graph now includes auxiliary biological nodes. This directly quantifies the value of heterogeneous context, independent of any relational parameterisation. Proteins, diseases, and side effects are carrying real and measurable signal for DDI prediction.

HGT pushes accuracy to 0.9181 but its macro F1 of 0.6185 is actually lower than heterogeneous GCN. Despite its type-aware attention mechanism, HGT does not translate this sophistication into better macro performance. Attention modulation over a shared parameter space appears less effective than fully separate transformations when the relation types carry fundamentally different biological semantics—a drug binding to a protein and a drug being associated with a disease are not variations on the same theme, and conflating them during aggregation loses information that RGCN preserves.

RGCN achieves the strongest performance across all metrics—accuracy 0.9521, macro recall 0.7196, and macro F1 0.6940. Relation-specific weight matrices are the right inductive bias for this problem. Each edge type in the heterogeneous graph represents a qualitatively different biological relationship, and learning entirely separate transformations per relation allows the model to preserve those distinctions through every layer of message passing.

The Hybrid RGCN-HGT model, evaluated as an ablation, achieves a macro F1 of 0.6785—lower than RGCN alone despite its additional complexity. The fusion layer that combines RGCN and HGT branch outputs introduces parameters that need to be optimised from a training signal that is already sparse for tail classes. Rather than complementing RGCN’s relational specificity, the HGT attention branch appears to dilute it during fusion, particularly for underrepresented interaction types where the gradient signal is weakest. This result makes a practical point—architectural complexity does not automatically translate to better performance in severely imbalanced multi-class settings. A well-configured single model with the right inductive bias outperforms a more complex hybrid that lacks a principled way to reconcile competing representational strategies under a skewed label distribution.

### 3.2 Hyperparameter tuning

To identify the optimal RGCN configuration, a systematic hyperparameter search was conducted, evaluating hidden dimension, number of layers, dropout rate, and learning rate independently with all other settings held constant. Results are summarised in Table 3.

**Table 3:** Hyperparameter search results for RGCN.

Hyperparameter	Values Tested	Best Value	Best Macro F1
Hidden Dimension	128, 192, 256, 384	**384**	**0.7108**
Num Layers	2, 3, 4, 5	**3**	**0.7051**
Dropout	0.1, 0.2, 0.3, 0.4	**0.2**	**0.7096**
Learning Rate	0.0005, 0.001, 0.002	**0.001**	**0.7108**

All other settings held constant during each sweep. Final model uses hidden dim 384, 3 layers, dropout 0.2, and lr 0.001. Best value per hyperparameter is highlighted in bold.

Hidden dimension 384 yields the best macro F1 of 0.7108, with performance improving consistently as capacity increases. Three layers proves optimal for depth—performance declines at 4 and 5 layers, consistent with the oversmoothing effect observed in deeper GNNs where node representations become increasingly indistinguishable with additional aggregation hops. A dropout rate of 0.2 strikes the best regularisation balance, and a learning rate of 0.001 provides stable convergence across the imbalanced label distribution. This final configuration—hidden dim 384, 3 layers, dropout 0.2, lr 0.001—is used for all subsequent experiments.

### 3.3 Class imbalance handling

Table 4 reports the performance of three class imbalance strategies applied to RGCN on the 105—class DDI prediction task.

**Table 4:** Performance comparison of class imbalance handling strategies.

Strategy	Accuracy	Precision	Recall	F1
Cross—Entropy	0.9559	0.7018	0.7326	0.7025
Weighted CE	0.9526	0.7033	0.7409	0.7070
Weighted Random Sampler	0.9266	0.6165	0.8222	0.6854
Tail—Aware Focal Loss	0.9488	**0.7070**	**0.8062**	**0.7341**

All experiments use RGCN as the base architecture with the optimal hyperparameter configuration.

Best results are highlighted in bold.

Standard cross—entropy achieves a macro F1 of 0.7025 and recall of 0.7326, serving as the baseline for all comparisons. Weighted cross-entropy produces a modest but consistent improvement—F1 rises to 0.7070 and recall to 0.7409—at essentially no additional computational cost. It is a useful baseline correction but not sufficient on its own for a distribution as severely skewed as this one.

Weighted random sampling produces the most uneven result. Recall climbs to 0.8222—the highest of any strategy—but precision collapses to 0.6165 and F1 drops to 0.6854, below the unweighted baseline. Oversampling rare classes forces the model to see them more frequently, but the head classes are undersampled in the process. The result is a model that detects more tail interactions but mispredicts a substantial number of head ones. High recall with collapsed precision is not clinically useful—it produces too many false positives to be reliable in practice.

Tail-Aware Focal Loss achieves the best overall balance. Recall reaches 0.8062 - nearly matching weighted random sampling—while precision holds at 0.7070 and F1 rises to 0.7341, the highest of all strategies. The focal modulation concentrates gradient signal on hard examples without starving the head classes, and the effective sample size weighting provides a smoother and more stable frequency correction than either raw reweighting or oversampling alone. This combination proves particularly well suited to the long-tailed 105 - class DDI distribution, where some tail classes have fewer than ten training examples and standard objectives provide essentially no useful gradient signal.

## 4 Conclusion

This work presents a large—scale heterogeneous biomedical graph and a systematic evaluation of GNN architectures and class imbalance strategies for fine—grained DDI prediction across 105 interaction classes. RGCN with relation—specific weight matrices consistently outperforms attention—based and homogeneous alternatives, demonstrating that relational inductive bias is the critical factor in heterogeneous DDI modelling. Tail—Aware Focal Loss, combining focal modulation with effective sample size weighting, achieves the best balance between head and tail class performance—a combination that matters clinically, where rare interactions carry disproportionate patient safety risk. The hybrid ablation further confirms that architectural complexity alone does not guarantee improvement under severely skewed label distributions. Together, these findings offer practical guidance for building DDI prediction systems that are both accurate and comprehensive across the full interaction space.

This study focuses on the transductive DDI prediction setting, where the objective is to infer missing interaction types between drugs already represented within the heterogeneous graph. This setting remains clinically relevant because many approved drugs possess extensive biological context but incomplete interaction annotations. An inductive evaluation involving entirely unseen drugs was not performed at this stage, as it would require additional constraints on feature availability, graph connectivity, and cold-start representation learning beyond the scope of this study. Extending the framework to zero-shot and inductive DDI prediction remains an important direction for future work.

## Data Availability

Code and data are available at https://github.com/VarshaGnanasekar/DDI_PREDICTION
